# Perfusion and pulsatile pressure: their relationship with target organ damage in the African-PREDICT study

**DOI:** 10.1186/s12872-024-04071-y

**Published:** 2024-08-01

**Authors:** Donavan Rooi, Shani Botha-Le Roux, Yolandi Breet

**Affiliations:** 1 Hypertension in Africa Research Team (HART), North-West University, Private Bag X 1290, Potchefstroom, 2520 South Africa; 2 MRC Research Unit for Hypertension and Cardiovascular Disease, North-West University, Potchefstroom, South Africa

**Keywords:** Pulse pressure, Mean arterial pressure, Echocardiography, Carotid intima media thickness, Arterial stiffness, Retinal vessel calibres, Endothelial function

## Abstract

**Background:**

Hypertension is the leading risk factor for subclinical target-organ damage (TOD) and cardiovascular disease (CVD). Little is known about the relationship between different pressure measures and subclinical TOD, especially in young populations. We compared the strength of associations of subclinical TOD markers with perfusion and pulsatile pressure in young adults.

**Methods:**

A total of 1 187 young adults from the African-PREDICT study were included. Ambulatory mean arterial pressure (MAP) and pulse pressure (PP) was obtained. Markers of subclinical TOD were measured and included left ventricular mass index (LVMi), carotid intimamedia thickness (cIMT), carotidfemoral pulse wave velocity (cfPWV), central retinal arteriolar equivalent (CRAE) and albumin to creatinine ratio (ACR).

**Results:**

Measures of sub-clinical TOD (cIMT, cfPWV and CRAE), associated stronger with perfusion pressure (all *p* < 0.001) than pulsatile pressure in unadjusted models. Stronger associations were found between cfPWV (adjusted R^2^ = 0.26), CRAE (adjusted R^2^ = 0.12) and perfusion pressure (all *p* ≤ 0.001) than pulsatile pressure independent of several non-modifiable and modifiable risk factors.

**Conclusions:**

In young, healthy adults, perfusion pressure is more strongly associated with subclinical TOD markers than pulsatile pressure. These findings contribute to the understanding of the development of early cardiovascular changes and may guide future intervention strategies.

## Introduction

Hypertension (HTN) has become a global burden. [1] In fact, South Africa is amongst countries that has the highest prevalence of HTN [2]. More recently, non-invasive technology has afforded the 24-hour assessment of both steadystate and pulsatile aspects of blood pressure (BP). [3] Technological advancement also allows for the comfortable and accurate measurement of subclinical targetorgan damage (TOD). [4] Subclinical TOD can be defined as an intermediate endpoint that signifies pathological changes occurring early-on within the myocardium, arteries and major organs and precedes hypertensive and atherosclerotic cardiovascular disease (CVD) development. [5] These structural and functional changes can encompass left ventricular (LV) hypertrophy, vascular wall remodelling, early renal dysfunction and retinopathy. [5, 6] Technological improvements in biological imaging and increased sensitivity of screening tests are making it apparent that people with milder BP elevations and who are asymptomatic, already present with subclinical TOD. [7]

Studies investigating the association between different measures of BP and TOD have yielded controversial results. [8] Comparative analyses between pulsatile pressure (pulse pressure (PP)) and perfusion pressure (mean arterial pressure (MAP)) are limited. Pulse pressure has been associated with CVD [9] but limited and conflicting research exists regarding MAP and CVD. [10, 11] In a prospective study of 11 150 male participants, with no history of CVD, there was a differential finding that MAP predicted CVD in younger men only, whereas PP predicted CVD in older men in the same sample. [12] Various studies showed correlations between certain pressure measures and markers of subclinical TOD, but the study cohorts were older than 30 years with existing pathology. [11, 13] Therefore, this study aimed to compare the strength of associations between measures of subclinical TOD with ambulatory MAP and PP in young, apparently healthy South African adults. These findings may give insights into the pathophysiology of subclinical TOD that precedes HTN and may highlight some of the underpinning pathophysiological mechanisms involved in early CVD development. Identifying and understanding these mechanisms may guide future interventions to combat the burden of HTN and CVD.

## Materials and methods

This study was based on the African Prospective study on the Early Detection and Identification of Cardiovascular Disease and Hypertension (African-PREDICT). [14] The African-PREDICT study is an on-going study that recruited 1202 white and black participants between the ages of 20–30 years from communities in and around the city of Potchefstroom, in the JB Marks local municipality, North West Province in central South Africa. The baseline recruitment for the African-PREDICT study took place from 2013 to 2017 and aims to investigate the underpinning pathophysiological mechanisms involved in CVD as well as predictors of CVD in an apparently healthy, South African, young adult population. Prospective participants were screened and those who met the criteria were included in the study. The inclusion criteria were young (20–30 years), apparently healthy, black and white men and women, with a normal screening clinic BP (< 140/90 mmHg), who were not infected with human immuno-deficiency virus (HIV), had no self-reported chronic disease (cancer, tuberculosis, liver disease, renal disease, diabetes and CVD) or medication use for chronic disease. Women that were pregnant or breastfeeding at the time of screening were excluded. For this study, participants with missing data for ambulatory MAP, PP and urinary ACR (*n* = 15) were excluded and therefore, a total of 1 187 participants were included.

Both the original African-PREDICT study and this sub-study were approved by the North-West University Health Research Ethics Committee with endorsement from the National and Provincial Department of Health. All procedures complied with the Declaration of Helsinki (2008) and conformed to the Medical Research Council guidelines of good clinical practice. All participants gave written informed consent prior to any measurements being performed.

### General health and demographic questionnaire

Self-reported participant demographics and lifestyle data were collected using a web-based questionnaire during a face-to-face session. These questionnaires were completed with the help of a research nurse, trained research assistant and trained postgraduate students. Socioeconomic status (SES) was calculated using a point system that was adapted from Kuppuswamy’s Socioeconomic Status Scale. [15] A SES score was calculated from highest level of education, skill-level and total household income using the information from the self-reported general health questionnaire. The SES score was then used to stratify the sample into SES classes namely: lower, middle and higher socioeconomic classes. [15]

### Anthropometric measurements

Trained anthropometrists measured weight (kg) to the nearest 0.01 kg, using an electronic body scale (SECA, Birmingham, UK) and height (cm) to the nearest 0.1 cm, using a stadiometer (SECA, Birmingham, UK). Waist circumference was measured three times using a non-flexible tape measure (Holtain, Crymych, UK) and recorded to the nearest 0.1 cm. The median of the three measurements was used in subsequent analyses. Body mass index (BMI) was calculated using the standard weight (kg)/height (m [2]) calculation. Anthropometric measurements were performed according to the International Society for the Advancement of Kinanthropometry guidelines. [16]

### Cardiovascular measurements

#### Ambulatory blood pressure

Participants were fitted with a validated 24-hour ambulatory BP apparatus (CardioXplore^®^ CE120, Meditech, Budapest, Hungary), programmed to take recordings every 30 min during the day (06h00 to 22h00) and every hour during the night (22h00 to 06h00). The ambulatory blood pressure apparatus was fitted to each participant at approximately the same time every day (late morning), using an appropriately sized cuff, fitted on the non-dominant arm. Mean systolic blood pressure (SBP), DBP and PP was recorded over the ambulatory period and the MAP was calculated using a formula. Mean arterial pressure was calculated as MAP = [diastolic BP] + 0.412 [pulse pressure]. [17] In determining if an ABPM measurement is successful there was a threefold requirement. Ambulatory BP measurements were considered successful with > 21 inflations during the day, > seven inflations during the night and finally a successful inflation rate of > 70%. In line with European Society of Hypertension practice guidelines. [18] The average successful cuff inflation rate for this study sample was 83%.

#### Carotid-femoral pulse wave velocity

The SphygmoCor XCEL device (SphygmoCor XCEL, AtCor by CardieX Ltd, Syndey, Australia) was used to measure carotid-femoral pulse wave velocity (cfPWV). The cfPWV was captured at the right carotid and femoral arterial pulse points. The participants were in a resting state and were placed in a supine position for the measurement to be done. The femoral arterial wave form was captured via an appropriately sized cuff placed around the right thigh and the right carotid arterial waveform was captured simultaneously via applanation tonometry. The distances between the pulsated sites (carotid-to-cuff with an infantometer, and femoral-to-cuff via a tape measure) were measured and 80% of these distances were used as the pulse wave travelled distance. [19] Carotid-femoral pulse wave velocity was automatically calculated as $\:\frac{distance}{pulse-transit\:time}$. The measurement was performed twice and repeated if the two measurements differed by more than 0.5 m/s. The averages of the two measurements that are within 0.5 m/s and three mmHg central SBP, of each other, were taken.

#### Carotid intima-media thickness

A trained sonographer obtained the data, and analysis of the images obtained was performed by an experienced registered clinical technician. Images were obtained from the left and right common carotid arteries with the General Electric Vivid E9 (GE Vingmed Ultrasound A/S, Horten, Norway) apparatus. Images were imported into Artery Measurement Systems software, which was then used to analyse the images (Gustavvson, Sweden). [20] Three optimal angles were captured per side, namely three angles on the right and three angles on the left common carotid artery, after which the average of the three angles was calculated to determine the thickness of the respective side. In this study, the average of the near and far wall carotid intima media thickness (cIMT) measurement was used.

#### Left ventricular mass index

A standard transthoracic echocardiography procedure was followed by a medical clinical technologist while each participant was in a partial left decubitus position with the head of the examining table modestly elevated. The General Electric Vivid E9 device (GE Vingmed Ultrasound A/S, Horten, Norway) was used along with the 2.5 to 3.5 MHz transducer and a single ECG-lead for timing purposes. Standardised methods were employed to obtain high quality recordings according to the current recommendations as outlined in the guidelines of the American Society of Echocardiography. [21] One trained sonographer obtained the data and the data was analysed by one experienced registered clinical technician. To determine the LVMi, left ventricular mass was calculated by a standard formula and normalised for both body surface area and height. [22]

#### Retinal microvascular measurements

Prior to the measurement, the research nurse determined the intraocular pressure in each eye with a Tonopen Avia (Reichert technologies) and Ocu-film sleeve. A local anaesthetic drop, Novesin Wander (0.4% eye drop), was applied to the eye before performing this measurement. If the eye pressure exceeded 24 mmHg, no further retinal measurements were done. Fifteen to 30 min prior to the retinal measurement, a drop of Tropicamide (1% Alcon) was administered in the right eye to induce mydriatic conditions. To avoid inducing angle-closure glaucoma, an estimation of the depth of the angle of the anterior chamber was done by the research nurse prior to administering the Tropicamide.

Retinal photography was performed using the Dynamic Retinal Vessel Analyzer (Imedos, Jena, Germany) fitted with a Zeiss Fundus camera FF-450. These images were used to evaluate retinal artery and vein calibres. Monochrome and colour retinal images were captured (using Visualis 2.81 software) at a 50° camera angle. The monochrome or colour image underwent vessel analysis using VesselMap2 software. All vessels located between one and 1.5 optic disc diameters from the outer margin of the optic disc were marked as either arteries or veins. The central retinal arteriolar equivalent (CRAE) was subsequently calculated using the Knudtson formula, where only the six largest arteriolar segments were included in the calculation. [23]

### Biological sampling and biochemical analyses

#### Spot-urine and blood sampling

Early in the morning, venous blood and spot-urine samples were collected. Samples were taken immediately to the on-site laboratory. The blood was centrifuged, and both the spot-urine and blood samples were aliquoted into cryovials for storage in bio-freezers at -80 °C. The blood sample tubes were of stabilyte, citrate, serum, ethylenediaminetetraacetic acid (EDTA), and sodium fluoride (NaF) and were centrifuged. All the cryovials centrifuged within 30 min after being collected.

### Biochemical analyses

Serum cotinine was analysed using a chemiluminescence method on the Immulite apparatus (Siemens, Erlangen, Germany). The Cobas Integra^®^ 400 plus (Roche, Basel, Switzerland) was used to measure urinary albumin by means of ion-selective electrode potentiometry. Urinary samples were analysed for albumin and creatinine (Cobas Integra^®^ 400plus, Roche, Basel, Switzerland) and the albumin-creatinine ratio (ACR) was calculated as albumin/creatinine. Serum samples were analysed for high-sensitivity C-reactive protein, total cholesterol, lowdensity lipoprotein cholesterol, high-density lipoprotein cholesterol and gamma-glutamyl transferase (Cobas Integra^®^ 400plus, Roche, Basel, Switzerland) and for IL-6 the high sensitivity Quantikine ELISA kit (R&D systems) was used. Glucose was analysed in NaF plasma using the UniCel^®^ DxC 800 Synchron^®^ Clinical System (Beckman Coulter, Indianapolis, US). Reactive oxygen species were determined in serum with highthroughput spectrophotometric assay by the Synergy H4 hybrid microplate reader (BioTek, Winooski, VT, USA). [24]

### Statistical analyses

Statistical analyses were performed with IBM^®^ SPSS^®^ Statistics version 27 software (IBM Corporation; Armonk, New York, USA). GraphPad Prism version 5.03 (GraphPad Software Inc., CA, USA) was used for the graphical illustration of data. Continuous variables were evaluated for normality by visual inspection (QQ plots) and non-Gaussian variables were transformed to the natural logarithm. Continuous data with a normal distribution were presented as arithmetic mean ± standard deviation and categorical data as proportions. Logarithmically transformed variables are presented as geometric means with their 5th and 95th percentile boundaries. Pearson and multiple regression analyses were performed to determine the associations between the dependent variables (ACR, LVMi, cIMT, cfPWV, CRAE) and independent variables (MAP and PP). Results were considered statistically significant when *p* ≤ 0.05. A backward stepwise linear regression was used to explore the independent association between measures of subclinical TOD and pulsatile- as well as perfusion pressure components, while controlling for the following predictors: age, sex, ethnicity, socioeconomic status score, low-density lipoprotein cholesterol, interleukin-6, reactive oxygen species, gamma-glutamyl transferase and cotinine. The predictors were determined by performing Pearson correlations with the main dependent and independent variables. The probability of F was set at 0.1.

Additionally, the Williams’ t-test, also referred to as the Student’s T Test, was used to compare the strength of correlation coefficients. To compare the strength of regression coefficients, a z-test was used to calculate p-values from z-values. [25]

## Results

Table 1 illustrates the general characteristics of the study sample. The median age of participants was 24 years (interquartile range: 5). The sex distribution comprised 52.2% women and 49.9% were of black ethnicity. Both 24 h MAP (88 ± 6.62) and PP (48 ± 7.26) were within normal ranges for the sample age.

**Table 1:** Characteristics of the study sample

*N*	1187
Women, N (%)	620 (52.2)
Black ethnicity, N (%)	592 (49.9)
Age (years)	24.6 ± 3.12
Socioeconomic status class	
Lower (n, %)	471 (39.6)
Middle (n, %)	340 (28.6)
Higher (n, %)	375 (31.6)
*Anthropometric measurements*	
Height (cm)	168 ± 9.55
Weight (kg)	69.4 (48.9; 103)
Body mass index (kg/m^2^)	24.5 (18.0; 35.5)
Waist circumference (cm)	79.3 (63.8; 103)
*Cardiovascular measurements*	
24 h Mean arterial pressure (mmHg)	88 ± 6.62
24 h Pulse pressure (mmHg)	48 ± 7.26
24 h Systolic blood pressure (mmHg)	117 ± 9.45
24 h Diastolic blood pressure (mmHg)	69 ± 5.89
Left ventricular mass index (g/m^2^)	75.8 ± 17.9
Carotid intima-media thickness (mm)	0.46 ± 0.04
Pulse wave velocity (m/s)	6.35 ± 0.94
Central retinal artery equivalent (M/U)	160 ± 11.8
*Biochemical markers*	
Urinary albumin-creatinine ratio (mg/mmol)	0.50 (0.16; 2.41)
Total cholesterol (mmol/L)	3.58 (2.00; 5.81)
High-density lipoprotein cholesterol (mmol/L)	1.16 ± 0.42
Low-density lipoprotein cholesterol (mmol/L)	2.26 (1.09; 4.19)
Triglycerides (mmol/L)	0.72 (0.32; 1.82)
Glucose (mmol/L)	4.10 ± 1.07
C-reactive protein (mg/L)	0.89 (0.08; 9.60)
Interleukin-6 (pg/ml)	1.08 (0.38; 3.77)
Reactive oxygen species (mmol/L)	47.5 ± 32.4
γ-glutamyl transferase (U/L)	18.3 (6.00; 54.8)
Cotinine* (ng/ml)	200 ± 140

Data expressed as arithmetic mean ± SD or geometric mean with 5th and 95th percentiles

*Only for participants with detectable levels of ≥ 10 ng/ml (n= 302)

**Fig. 1:**
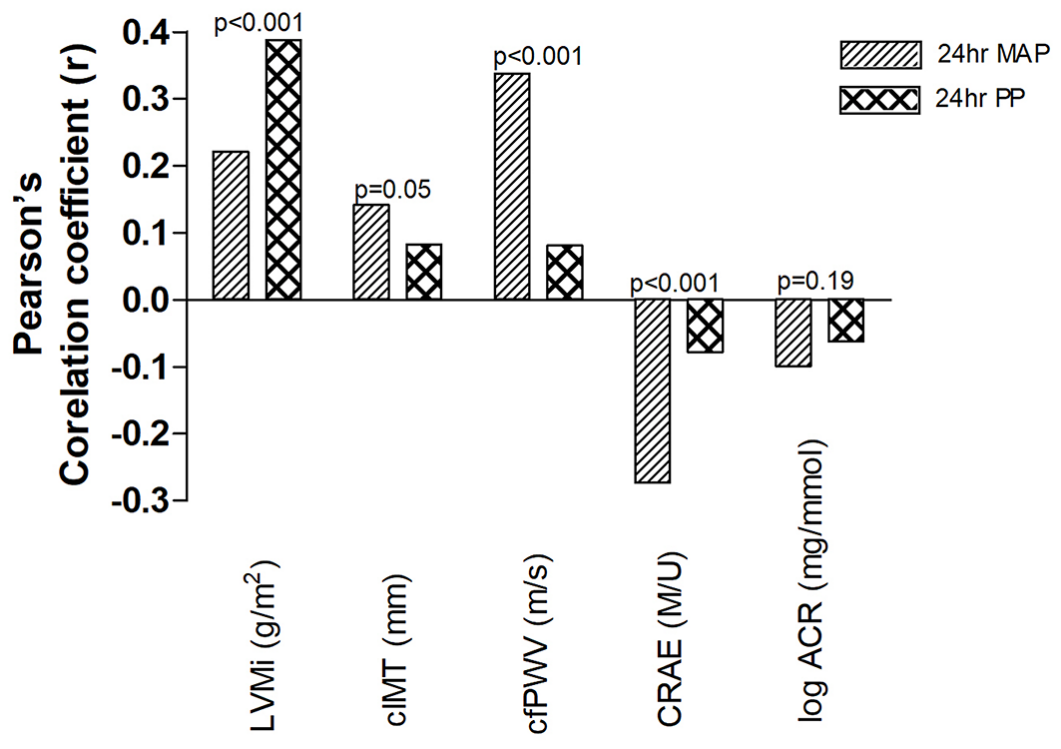
Comparing the strength of correlations between different subclinical target organ damage markers with perfusion and pulsatile pressure, respectively. All individual correlations were statistically significant. 24 h MAP: 24-hour mean arterial pressure; 24 h PP: 24-hour pulse pressure; ACR: albumin-to-creatinine ratio; LVMi: left ventricular mass index; cIMT: carotid intima media thickness of the near wall; cfPWV: carotid-femoral pulse wave velocity; CRAE: central retinal arteriolar equivalent

In Fig. 1, the strength of the relationships of 24 h MAP and PP with the different subclinical TOD markers were compared. Left ventricular mass index correlated positively with PP (*r* = 0.39, *p* < 0.001), which was stronger (*p* < 0.001) than the relationship with MAP (*r* = 0.22, *p* < 0.001). Carotid intima-media thickness correlated positively with both MAP (*r* = 0.14, *p* < 0.001) and PP (*r* = 0.11, *p* < 0.004), but stronger with MAP (*p* = 0.05). The positive correlation between cfPWV and MAP (*r* = 0.34, *p* < 0.001) was stronger (*p* < 0.001) than with PP (*r* = 0.08, *p* = 0.006). A negative correlation was found between CRAE and MAP (*r*= -0.27, *p* < 0.001), which was stronger (*p* < 0.001) than with PP (*r*= -0.10, *p* = 0.011). The ACR also negatively correlated with both MAP (r_s_ = -0.10, *p* = 0.001) and PP (r_s_ = -0.06, *p* = 0.035), but the strength of these associations was comparable (*p* = 0.19).

**Fig. 2:**
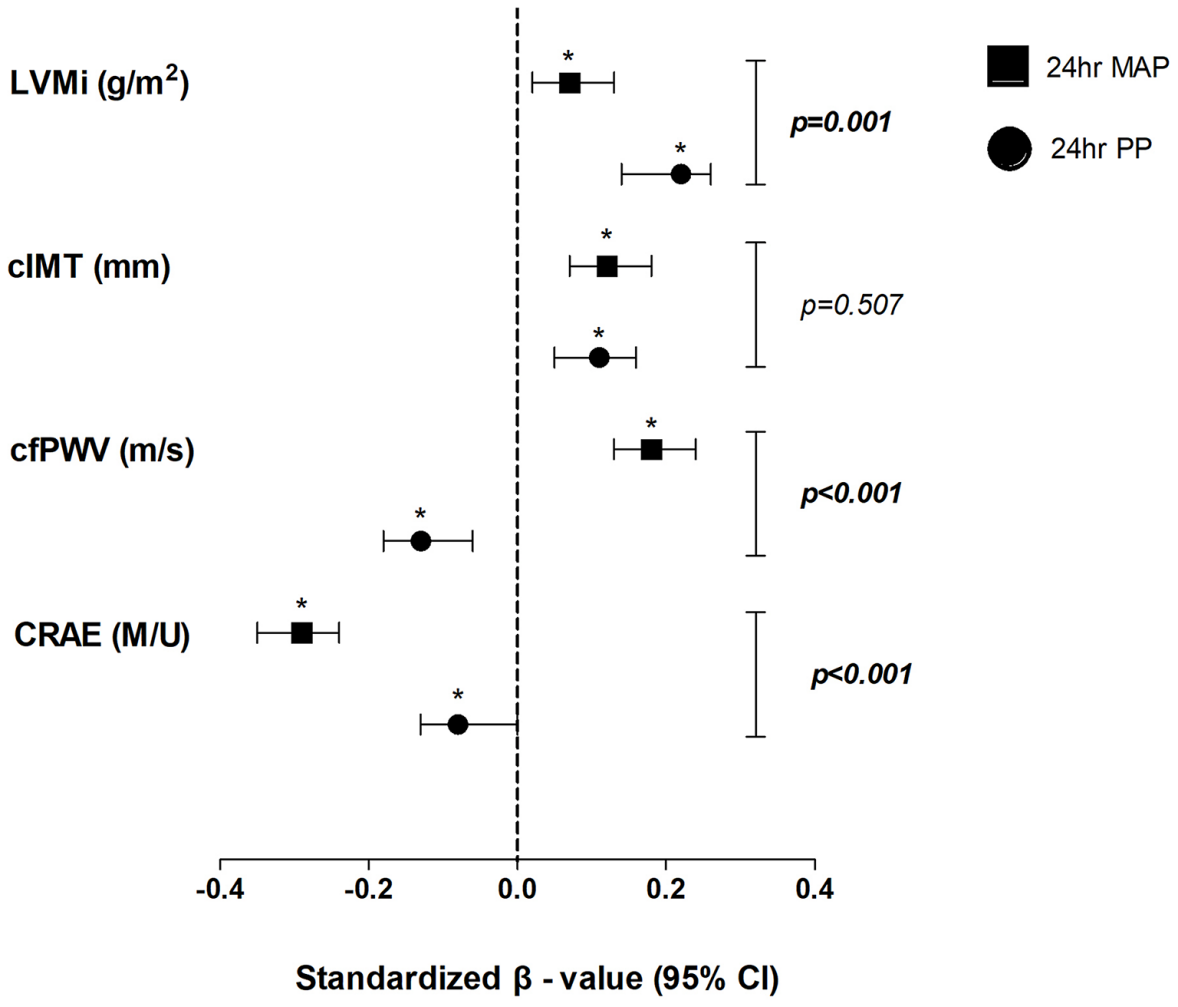
Independent relationships between markers of TOD and perfusion and pulsatile measures, respectively. (*)Indicates statistically significant associations (*p* < 0.05) between outcome measures and mean arterial pressure or pulse pressure. P-values indicate statistical differences in strength of associations. Confounding variables included in the models: age, sex (men), ethnicity (white), socioeconomic status score, low-density lipoprotein, interleukin-6, reactive oxygen species, gamma-glutamyl transferase, and cotinine. 24 h MAP: 24-hour mean arterial pressure; 24 h PP: 24-hour pulse pressure; LVMi: left ventricular mass index; cIMT: carotid intima media thickness

In Fig. 2 independent relationships between standardized markers of TOD and 24-hour perfusion and pulsatile pressure, respectively, was performed. Associations were found between all individual TOD measures (LVMi, cIMT, cfPWV, and CRAE) with both pressure measures (MAP and PP) (all *p* < 0.05). In terms of perfusion pressure, left ventricular mass index (Adjusted R^2^ = 0.22, β = 0.07) and IMT (Adjusted R^2^ = 0.04, β = 0.13) associated positively with MAP. An unexpected finding was a negative association (Adjusted R^2^ = 0.24, β = -0.13; *p* < 0.001) of cfPWV with pulsatile pressure. Furthermore, we compared the strength of the regression coefficients of MAP versus PP and found that the associations of cfPWV and CRAE were strongest with MAP (*p* < 0.001) and LVMi strongest (*p* = 0.001) with PP. Urinary ACR did not enter the backward stepwise regression models and hence was not used in the comparison of the regression coefficients.

## Discussion

In this study, the aim was to compare the strength of associations between perfusion and pulsatile pressure measures with markers of subclinical TOD in young South African adults. The most prominent finding was that carotid wall thickness, arterial stiffness and narrower retinal arteriolar had stronger associations with perfusion pressure than with pulsatile pressure. However, after adjusting for confounders, this was not the case for carotid wall thickness. The LV hypertrophy marker had a stronger positive association with pulsatile pressure than with perfusion pressure. This remained the case after adjusting for confounding factors. An unexpected finding was a significant negative association of ACR with perfusion and pulsatile pressure. However, there was no significant difference in the association of ACR with the pressure measures.

The stronger independent association of MAP with markers of arterial stiffness and retinal arteriolar narrowing suggests that perfusion pressure may have a more substantial contribution to subclinical TOD development in this young adult population. This finding supports a previous study, with a median 10.8 year follow-up, which determined that MAP has a predictive role for CVD in men younger than 60 years. [12] The Framingham Heart Study suggested that abnormalities in perfusion pressure, that leads to increased cardiac output, have a predominant effect on BP in young adults. A separate publication by the Framingham heart study, consisting of 2 036 participants, showed that there was a linear increase of MAP from age 30. However, MAP plateaued after age 50 to 60 after which PP rose sharply. [26] The sharp rise of PP after age 50 implies that PP may not be a sensitive predictor of risk in young populations. As the findings of this study suggest that MAP is associated with arterial stiffness and retinal arteriolar narrowing, it may be important to investigate the contribution of MAP to cardiovascular risk stratification, especially in young adults, to prevent early CVD development. Early elevation of MAP is indeed a manifestation of increased cardiac output or vascular peripheral resistance, possibly due to a myriad of factors such as activation of the sympathetic nervous system, hypervolemia, small vessel disease or vascular dysfunction. [27] These findings remained robust after taking several risk factors into account.

The stronger negative association of LVMi with PP, rather than MAP, is not aligned with previous findings that both MAP and central PP was positively related to LVMi. [28] That is, an increase in MAP and PP leads to increased LV hypertrophy because concentric LV hypertrophy is associated with increased arterial stiffness, and decreased arterial compliance in young adults. [29] Further investigation is required to clarify this finding.

It was also found that there was a negative association of cfPWV with pulsatile pressure after adjustments. This is an unexpected finding as cfPWV has been demonstrated to be a major determinant of PP. [30] Emerging research suggests there may be a pressure-independent relationship between peripheral sympathetic out flow and central aortic PWV in young and healthy individuals at rest. [31] Therefore, acute elevations in peripheral sympathetic activity can increase central aortic PWV in young participants independent of a change in PP or heart rate. [32] However, as the ambulatory blood pressure was done on the non-dominant arm (left arm) and the cfPWV was done on the right thigh and right carotid site according to guidelines and recommendations, this may be a factor to consider.

An unexpected finding in the study was a negative association of ACR with MAP and PP with no difference in strength of association of ACR between the pressure measures. In contrast to this study’s findings, other research showed that there was a significant positive correlation between ACR and MAP. [33] It has also been suggested that PP may play a role in the development of renal damage. [34] When kidney function is optimal, only metabolic waste products are filtered into the urine, signifying healthy glomerular podocyte function. [35] Therefore, it is expected that, when MAP and PP are elevated, the ACR will also increase, denoting a positive correlation. [35] The reason for the negative correlation between BP measures and ACR in this study is unclear, as it is unlikely that glomerular and vascular endothelial dysfunction will be present at this young age. [35] This warrants further investigation, especially within a longitudinal setting.

Few studies have compared the strength of association of MAP and PP with CVD. One study investigated office brachial SBP, DBP, MAP and PP as independent predictors of CVD, all-cause and CVD mortality. The study had 5 991 participants aged ≥ 30 years without baseline CVD and antihypertensive medication. It was found that, in participants aged < 60 years, MAP and PP were associated with total mortality. [36] However, in participants aged ≥ 60 years, only SBP and PP increased the risk of all-cause mortality, implying once more that PP may be a more sensitive measure than MAP, for CVD in older populations. In a similar prospective study that followed 11 150 male participants with no history of CVD or antihypertensive treatment, MAP was associated with CVD in younger men, [12] thus echoing the finding of stronger association of MAP with subclinical TOD in the participants.

This study sample consisted of apparently healthy young adults. All blood pressure measures, and markers of TOD were within normal ranges. As this study focused on young and apparently healthy participants, it should be noted that the positive association of the pressure measures with subclinical TOD does not imply adverse end-organ damage yet, but rather represents that the positive association may imply pathophysiological development towards future end-organ damage.

Some strengths and limitations should be taken into consideration when interpreting the results from this study. When compared to previous studies [11, 13, 28] this study focused on five different subclinical TOD markers, the most out of previous investigations. Another strength of the study is the use of 24 h BP instead of office BP. As the study sample consisted of participants from specific urban areas of South Africa, the findings may not be representative of the whole population. Due to the cross-sectional study design of the study, causality could not be inferred. The African-PREDICT study, of which the current study is a sub-study, is prospective and may provide more insight into the strength of association of perfusion and pulsatile pressure with subclinical TOD over time.

In conclusion, markers of subclinical TOD, including carotid wall thickness, arterial stiffness and retinal arteriolar narrowing showed stronger associations with perfusion pressure than with pulsatile pressure in a young adult sample. In low to middle-income countries, such as South Africa, with a high prevalence of HTN and CVD, these findings are important as MAP is often overlooked when screening for cardiovascular risk.

## Data Availability

The data that support the findings of this study are not openly available due to reasons of sensitivity and are available from the corresponding author upon reasonable request. Data are located in controlled access data storage at North-West University, South Africa.

## References

[1] Lackland DT, Weber MA. Global burden of cardiovascular disease and stroke: hypertension at the core. Can J Cardiol. 2015;31(5):569–71. 10.1016/j.cjca.2015.01.009. https://doi.org/https://doi.org/. 25795106 10.1016/j.cjca.2015.01.009

[2] Gómez-Olivé X, Ali SA, Made F, Kyobutungi C, Nonterah E, Micklesfield L, et al. Regional and sex differences in the prevalence and awareness of hypertension: an H3Africa AWI-Gen study across 6 sites in sub-saharan Africa. Glob Heart. 2017;12(2):81–90. 10.1016/j.gheart.2017.01.007.PMC5967381. 28302553 10.1016/j.gheart.2017.01.007.PMC5967381 PMC5967381

[3] Omboni S, Posokhov I, Kotovskaya Y, Protogerou AD, Blacher J. Twenty-four‐hour ambulatory pulse wave analysis in hypertension management: current evidence and perspectives. J Curr Hypertens Rep. 2016;18(10):72. 10.1007/s11906-016-0681-2. 10.1007/s11906-016-0681-2 27659178

[4] Omboni S, Posokhov I, Parati G, Rogoza A, Kotovskaya Y, Arystan A, et al. Ambulatory blood pressure and arterial stiffness web-based telemonitoring in patients at cardiovascular risk. First results of the VASOTENS (vascular health ASsessment of the hypertENSive patients) Registry. J Clin Hypertens. 2019;21(8):1155–68. 10.1111/jch.13623. 10.1111/jch.13623 PMC8030443 31294910

[5] Devereux RB, Alderman MH. Role of preclinical cardiovascular disease in the evolution from risk factor exposure to development of morbid events. Circulation. 1993;88(4):1444–55. 10.1161/01.cir.88.4.1444. 8403291 10.1161/01.cir.88.4.1444

[6] Nadar SK, Tayebjee MH, Messerli F, Lip G. Y. Target organ damage in hypertension: pathophysiology and implications for drug therapy. Curr Phar Des. 2006;12(13):1581–892. 10.2174/138161206776843368. 10.2174/138161206776843368 16729871

[7] Williams B, Mancia G, Spiering W, Agabiti Rosei E, Azizi M, Burnier M, et al. 2018 ESC/ESH guidelines for the management of arterial hypertension. Eur Heart J. 2018;39(33):3021–104. 10.1093/eurheartj/ehy339. 30165516 10.1093/eurheartj/ehy339

[8] Bliziotis IA, Destounis A, Stergiou GS. Home versus ambulatory and office blood pressure in predicting target organ damage in hypertension: a systematic review and meta-analysis. J Hypertens. 2012;30(7):1289–99. 10.1097/HJH.0b013e3283531eaf. 22499289 10.1097/HJH.0b013e3283531eaf

[9] Domanski MJ, Davis BR, Pfeffer MA, Kastantin M, Mitchell GF. Isolated systolic hypertension prognostic information provided by pulse pressure. Hypertension. 1999;34(3):375–80. 10.1161/01.hyp.34.3.375. 10489379 10.1161/01.hyp.34.3.375

[10] Chen C, Ting C, Lin S, Hsu T, Ho S, Chou P, et al. Which arterial and cardiac parameters best predict left ventricular mass? Circulation. 1998;98(5):422–8. 10.1161/01.CIR.98.5.422. 9714092 10.1161/01.CIR.98.5.422

[11] Kong MG, Kim H, Kim M-A, Kim M, Park S, Yoon H, et al. Relationships between blood pressure measurements and target organ damage: data from the Korea women’s chest pain registry. J Clin Hypertens. 2018;20(12):1724–30. 10.1111/jch.13417. 10.1111/jch.13417 PMC8030790 30362256

[12] Sesso H, Stampfer M, Rosner B, Hennekens C, Gaziano J, Manson J, et al. Systolic and diastolic blood pressure, pulse pressure, and mean arterial pressure as predictors of cardiovascular disease risk in men. Hypertension. 2000;36(5):801–7. 10.1161/01.hyp.36.5.801. 11082146 10.1161/01.hyp.36.5.801

[13] Salvetti M, Agabiti Rosei C, Paini A, Aggiusti C, Cancarini A, Duse S, et al. Relationship of wall-to-lumen ratio of retinal arterioles with clinic and 24-hour blood pressure. Hypertension. 2014;63(5):1110–5. 10.1161/HYPERTENSIONAHA.113.03004. 24516107 10.1161/HYPERTENSIONAHA.113.03004

[14] Schutte AE, Gona PN, Delles C, Uys AS, Burger A, Mels CM, et al. The African prospective study on the early detection and identification of Cardiovascular disease and hypertension (African-PREDICT): design, recruitment and initial examination. Eur J Prev Cardiol. 2019;26(5):458–70. 10.1177/2047487318822354PMC6423686. 30681377 10.1177/2047487318822354PMC6423686 PMC6423686

[15] Patro BK, Jeyashree K, Gupta PK. Kuppuswamy’s socioeconomic status scale 2010-the need for periodic revision. Indian J Pediatr. 2012;79(3):395–6. 10.1007/s12098-011-0517-7. 21761123 10.1007/s12098-011-0517-7

[16] Stewart A, Marfell-Jones M, Olds T, De Ridder J. International standards for anthropometric assessment. Wellington, New Zealand: International Society for the Advancement of Kinanthropometry; 2011.

[17] Meaney E, Alva F, Moguel R, Meaney A, Alva J, Webel R. Formula and nomogram for the sphygmomanometric calculation of the mean arterial pressure. Heart. 2000;84:64. 10.1136/heart.84.1.64. 10862592 10.1136/heart.84.1.64 PMC1729401

[18] Stergiou GS, Palatini P, Parati G, O’Brien E, Januszewicz A, Lurbe E, et al. European Society of Hypertension practice guidelines for office and out-of-office blood pressure measurement. J Hypertens. 2021;39(7):1293–302. 10.1097/HJH.0000000000002843. 33710173 10.1097/HJH.0000000000002843

[19] Van Bortel LM, Laurent S, Boutouyrie P, Chowienczyk P, Cruickshank JK, De Backer T, et al. Expert consensus document on the measurement of aortic stiffness in daily practice using carotid-femoral pulse wave velocity. J Hypertens. 2012;30(3):445–8. 10.1097/HJH.0b013e32834fa8b0. 22278144 10.1097/HJH.0b013e32834fa8b0

[20] Touboul PJ, Hennerici MG, Meairs S, Adams H, Amarenco P, Bornstein N et al. Mannheim carotid intima-media thickness consensus (2004–2006). An update on behalf of the Advisory Board of the 3rd and 4th Watching the Risk Symposium, 13th and 15th European Stroke Conferences, Mannheim, Germany, 2004, and Brussels, Belgium, 2006. Cerebrovasc Dis. 2007;23(1):75–80. 10.1159/000097034 10.1159/000097034 17108679

[21] Lang RM, Badano LP, Mor-Avi V, Afilalo J, Armstrong A, Ernande L, et al. Recommendations for cardiac chamber quantification by echocardiography in adults: an update from the American Society of Echocardiography and the European Association of Cardiovascular Imaging. Eur Heart J Cardiovasc Imaging. 2015;16(3):233–70. 10.1093/ehjci/jev014. 25712077 10.1093/ehjci/jev014

[22] de Simone G, Kizer JR, Chinali M, Roman MJ, Bella JN, Best LG, et al. Normalization for body size and population-attributable risk of left ventricular hypertrophy: the strong heart study. Am J Hypertens. 2005;18(2 Pt 1):191–6. 10.1016/j.amjhyper.2004.08.032. 15752946 10.1016/j.amjhyper.2004.08.032

[23] Knudtson MD, Lee KE, Hubbard LD, Wong TY, Klein R, Klein BE. Revised formulas for summarizing retinal vessel diameters. Curr Eye Res. 2003;27(3):143–9. 10.1076/ceyr.27.3.143.16049. 14562179 10.1076/ceyr.27.3.143.16049

[24] Hayashi I, Morishita Y, Imai K, Nakamura M, Nakachi K, Hayashi T. High-throughput spectrophotometric assay of reactive oxygen species in serum. Mutat Res. 2007;631(1):55–61. 10.1016/j.mrgentox.2007.04.006. 17499011 10.1016/j.mrgentox.2007.04.006

[25] Clogg CC, Petkova E, Haritou A. Statistical methods for comparing regression coefficients between models. Am J Sociol. 1995;100(5):1261–93. 10.1086/230638. 10.1086/230638

[26] Franklin SS, Gustin W, Wong ND, Larson MG, Weber M, Kannel W, et al. Hemodynamic patterns of age-related changes in blood pressure: the Framingham Heart Study. Circulation. 1997;96(1):308–15. 10.1161/01.CIR.96.1.308. 9236450 10.1161/01.CIR.96.1.308

[27] Folkow B. Physiological aspects of primary hypertension. Physiol Rev. 1982;62(2):347–504. 10.1152/physrev.1982.62.2.347. 6461865 10.1152/physrev.1982.62.2.347

[28] Vasan RS, Short MI, Niiranen TJ, Xanthakis V, DeCarli C, Cheng S, et al. Interrelations between arterial stiffness, target organ damage, and cardiovascular disease outcomes. J Am Heart Assoc. 2019;8(14):e012141. 10.1161/JAHA.119.012141PMC6662123. 31303106 10.1161/JAHA.119.012141PMC6662123 PMC6662123

[29] Toprak A, Reddy J, Chen W, Srinivasan S, Berenson G. Relation of pulse pessure and arterial stiffness to concentric left ventricular hypertrophy in young men (from the Bogalusa Heart Study). Am J Cardiol. 2009;103(7):978–84. 10.1016/j.amjcard.2008.12.011. 19327426 10.1016/j.amjcard.2008.12.011

[30] Ni Y, Wang H, Hu D, Zhang W. The relationship between pulse wave velocity and pulse pressure in Chinese patients with essential hypertension. Hypertens Res. 2003;26(11):871–4. 10.1291/hypres.26.871. 14714577 10.1291/hypres.26.871

[31] Świerblewska E, Hering D, Kara T, Kunicka K, Kruszewski P, Bieniaszewski L, et al. An independent relationship between muscle sympathetic nerve activity and pulse wave velocity in normal humans. J Hypertens. 2010;28(5):979–84. 10.1097/hjh.0b013e328336ed9a. 20408258 10.1097/hjh.0b013e328336ed9a

[32] Nardone M, Incognito AV, Millar PJ. Evidence for pressure-independent sympathetic modulation of central pulse wave velocity. J Am Heart Assoc. 2018;7(3). 10.1161/JAHA.117.007971. 10.1161/JAHA.117.007971 PMC5850264 29378730

[33] Gilbert R, Phillips P, Jerums G. Relationship between ambulatory blood pressure and albuminuria in normal subjects. Am J Hypertens. 1991;4(12):959–62. 10.1093/ajh/4.12.959. 1815654 10.1093/ajh/4.12.959

[34] Viazzi F, Leoncini G, Parodi D, Ravera M, Ratto E, Vettoretti S, et al. Pulse pressure and subclinical cardiovascular damage in primary hypertension. Nephtol Dial Transpl. 2002;17(10):1779–85. 10.1093/ndt/17.10.1779. 10.1093/ndt/17.10.1779 12270984

[35] Deckert T, Feldt-Rasmussen B, Borch-Johnsen K, Kofoed-Enevoldsen TJ. Albuminuria reflects widespread vascular damage: the Steno hypothesis. Diabetologia. 1989;32(4):219–22. 10.1007/BF00285287. 2668076 10.1007/BF00285287

[36] Hadaegh F, Shahfiee M, Hatami M, Aziz F. Systolic and diastolic blood pressure, mean arterial pressure and pulse pressure for prediction of cardiovascular events and mortality in a Middle Eastern population. Blood Press. 2011;21(1):12–8. 10.3109/08037051.2011.585808. 21679012 10.3109/08037051.2011.585808

